# Mechanical Small Bowel Obstruction Secondary to Deflated and Migrated Intragastric Balloon

**DOI:** 10.5811/cpcem.64610

**Published:** 2026-09-26

**Authors:** Luis Moas, Arturo Feraud, Aliyah Baksh, Eric Boccio

**Affiliations:** * Mount Sinai Medical Center, Department of Emergency Medicine, Miami Beach, Florida; † Mount Sinai Medical Center, Department of Internal Medicine, Miami Beach, Florida; ‡ Florida International University, Herbert Wertheim College of Medicine, Department of Emergency Medicine, Miami, Florida

**Keywords:** intragastric balloon, small bowel obstruction, gastric balloon migration, clinical image

## Abstract

**Case presentation:**

A 29-year-old woman presented to the emergency department three months after intragastric balloon placement with nausea, vomiting, constipation, and abdominal pain. Computed tomography (CT) demonstrated spontaneous deflation and migration of the device into the small bowel with resultant mechanical small bowel obstruction. The patient was managed conservatively with bowel rest, intravenous fluids, electrolyte repletion, and nasogastric decompression. Repeat CT on hospital day 2 confirmed distal migration of the balloon into the descending colon with interval decompression of the small bowel. On hospital day 3, the patient reported spontaneous passage of the balloon per rectum with complete resolution of symptoms.

**Discussion:**

Spontaneous deflation and distal migration of an intragastric balloon causing small bowel obstruction is a rare but potentially life-threatening complication. Most reported cases require surgical or endoscopic intervention. This case highlights spontaneous transanal passage as a rare but favorable outcome achievable with conservative management in carefully selected, hemodynamically stable patients without signs of peritonitis, bowel ischemia or perforation, or failure of the intragastric balloon to progress.

## CASE PRESENTATION

A 29-year-old woman with obesity (body mass index, 32.8 kg/m^2^) presented to the emergency department (ED) with a two-day history of intractable nausea, bilious vomiting, constipation, and diffuse abdominal pain three months after undergoing intragastric balloon placement at a facility in Mexico. She reported a 35-pound reduction in weight during that time but had no follow-up with the physician who inserted the device. She endorsed up to seven episodes of nonbloody, bilious vomiting per day, intolerance of oral intake, and no bowel movements for three days.

The patient’s initial ED triage vital signs were within normal limits. Abdominal examination demonstrated mild distension with the point of maximal tenderness in the left lower quadrant; there was no rebound tenderness or guarding and bowel sounds were present. Laboratory evaluation was notable for hypokalemia (potassium, 3.3 mmol/L; reference range, 3.5–5.0 mmol/L), with an otherwise unremarkable complete metabolic panel, complete blood count, and lipase.

Noncontrast computed tomography (CT) of the abdomen and pelvis demonstrated a deflated and displaced intragastric balloon with associated bowel wall edema and a dilated loop measuring up to 3.2 cm, consistent with a developing small bowel obstruction (Images 1 and 2). General surgery and gastroenterology were consulted, and the patient was admitted and managed conservatively with nasogastric tube decompression, strict nothing per os status, maintenance intravenous fluid infusion, potassium repletion, and serial abdominal examinations. Repeat contrast-enhanced CT of the abdomen and pelvis on hospital day 2 demonstrated interval transit of the balloon into the descending colon with decompression of previously dilated small bowel loops and no evidence of perforation (Image 3). On hospital day 3, the patient reported spontaneous passage of the balloon per rectum with complete resolution of symptoms, and she was discharged in stable condition.

*CPC-EM Capsule*

What do we already know about this clinical entity?

*Intragastric balloon deflation and migration can cause rare, life-threatening small bowel obstruction, traditionally requiring surgical or endoscopic removal.*

What is the major impact of the image(s)?

*Serial computed tomography shows balloon migration from obstructing the small bowel to spontaneous passage through the colon.*

How might this improve emergency medicine practice?

*Conservative management with serial exams and imaging in stable patients with progressing balloon transit is feasible.*

## DISCUSSION

Intragastric balloon therapy has been used for weight loss since Nieben and Harboe introduced the procedure in 1982, offering a nonsurgical option for patients who do not qualify for bariatric surgery.^1^ Spontaneous deflation and distal migration causing small bowel obstruction is a rare complication estimated to occur in fewer than 1% of cases.^2^ Upon deflation, the collapsed silicone envelope may migrate through the pylorus and become impacted anywhere from the duodenum to the terminal ileum. Hay et al., in a review of 27 published cases, found that open or laparoscopic enterotomy and extraction was required in approximately 70% of cases, with the anatomical location of impaction guiding management.^2^ Endoscopic retrieval is feasible when the balloon is accessible—most reliably from the duodenum—although successful endoscopic retrieval from the jejunum and retrograde colonoscopic retrieval from the terminal ileum have also been reported.^3^–^4^ Timely removal is critical given the risk of progressive complications including bowel ischemia and perforation.

Conservative management without operative or endoscopic intervention is reasonable in hemodynamically stable patients with partial obstruction when spontaneous passage is anticipated.^5^ Surgical removal is generally recommended if symptoms do not improve or the balloon fails to progress within 48 hours.^6^ The specific intragastric balloon model, fill volume, and mechanism of deflation were unavailable because the procedure was performed outside the treating institution.

The present case demonstrates that spontaneous transanal passage is achievable with close clinical and radiographic monitoring and underscores the diagnostic value of serial abdominal examinations and CT imaging in guiding this decision. Emergency clinicians should maintain a high index of suspicion for mechanical small bowel obstruction in any patient with a history of intragastric balloon placement presenting with obstructive symptoms, regardless of time since placement, and a multidisciplinary approach that incorporates timely consultation with surgical and gastroenterological specialists remains essential.

## Figures and Tables

**Image 1:**
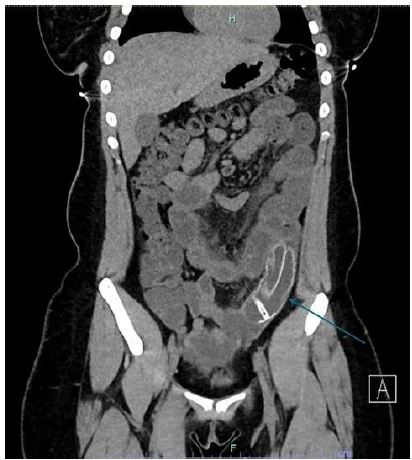
Coronal noncontrast computed tomography of the abdomen and pelvis obtained during the workup in the emergency department demonstrating the displaced intragastric balloon (arrow) within small bowel loops in the left lower quadrant consistent with a mechanical small bowel obstruction.

**Image 2:**
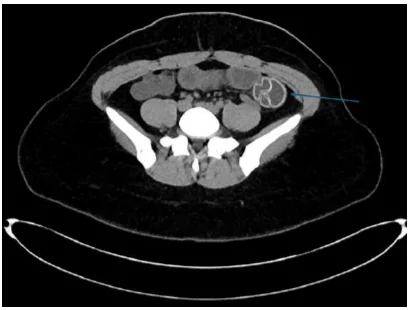
Axial noncontrast computed tomography of the abdomen and pelvis obtained during the workup in the emergency department demonstrating the displaced intragastric balloon (arrow) within the small bowel of the left lower quadrant.

**Image 3:**
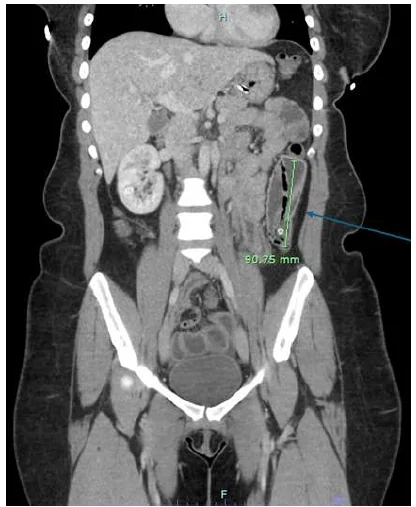
Coronal contrast-enhanced computed tomography of the abdomen and pelvis obtained on hospital day 2 demonstrating the intragastric balloon (9.1 cm in the craniocaudal dimension), positioned within the descending colon with interval resolution of the small bowel obstruction (arrow).

## References

[1] Nieben OG, Harboe H (1982). Intragastric balloon as an artificial bezoar for treatment of obesity. Lancet.

[2] Hay D, Ryan G, Somasundaram M (2019). Laparoscopic management of a migrated intragastric balloon causing mechanical small bowel obstruction: a case report and review of the literature. Ann R Coll Surg Engl.

[3] Bamakhrama K, Riyaz S, Musthafa M (2024). Small intestinal obstruction secondary to migrated intragastric balloon retrieved endoscopically from the jejunum. Eur J Case Rep Intern Med.

[4] Damin DC, Biazi BH, Colles T (2020). First colonoscopic management of a small-bowel obstruction secondary to intragastric balloon migration. Gastrointest Endosc.

[5] Ntyl S, Shalhoub M, AlShlwi S (2022). Small bowel obstruction secondary to gastric balloon migration: a case report. Int J Surg Case Rep.

[6] Molina GA, Palacios Gordon MF, Jerez Ortiz JR (2019). Bowel obstruction due to the migration of the deflated intragastric balloon, a rare and potentially lethal complication. J Surg Case Rep.

